# A New Tæniafuge

**Published:** 1886-10

**Authors:** Numa Campi, F. R. Campbell


### *A NEW TÆNIAFUGE.*

By NUMA CAMPI.

Translated from *Il Raccoglitore Medico*, by F. R. CAMPBELL, M. D.

The varieties of tænia described by authors are very numerous. We have only to open a book on zoology or parasitology to find a host of them described, with excellent illustrations. It is not my purpose to give a history of all the varieties of tape-worm, but I will content myself with the discussion of a single variety, the *tænia medio-canellata*, the one found in the modest field of my observations, and upon which alone I have tested the powers of a new remedy which has never before, to my knowledge, been employed as a tæniafuge.

This parasite, which so frequently affects mankind, producing the gravest disturbances, and even causing death, has been known from the remotest antiquity. We learn that Theophrastus and Dioscorides employed male fern to destroy it, that Vallisneri has seen Israelites and Egyptians affected with tape-worm, and that other distinguished observers have found it at Cairo, in Abyssinia, in South Africa, Egypt, Syria, and the New World.

The experiments of Perroncito and the observations of many physicians have shown that uncooked beef and mutton are the source of this *tænia medio-canellata* in man. Dr. Knok relates that the English engaged in the Caffre War, in 1819, subsisted largely on raw beef and that the soldiers of this expedition were affected with tænia to an alarming extent. The Russian physician, Kaschin, states that the Cossacks in some regions are affected with tape-worm almost to a man, because they do not properly cook the beef they consume. He states that among 500 persons received into the hospitals, not one was free from tape-worm. In Europe there is no region free from this parasite. In Italy it has become much more common since the use of raw or uncooked beef has become general.

The *tænia medio-canellata* or *tænia inerme* of man is larger, stronger, and more opaque than the *tænia solium.* It is the most difficult to expel of all the cestodes. The head is very large, and on it are located four large sucking discs without hooklets or rostella. The neck is quite short. The length of the body is ordinarily four or five metres; its segments or proglottides are thicker and longer than those of the *tænia solium.* The ova, as they come from the mature proglottides, are taken into the system of some animals and are developed into cysticerci. When these cysticerci are swallowed by man new tape-worms are produced. A peculiarity worthy of note is its extraordinary fecundity. It is generally admitted that from seventy to a hundred mature segments are found in this tape-worm at one time, each of these segments being filled with ova. According to the calculations of an English physician, a person harboring a tape-worm eliminates about 400 segments a month and consequently becomes the means of diffusing 100,000,000 of eggs a year. It is thus easy to understand that although the greatest majority of the ova perish, some will attain a complete development and thus secure to the species an extensive propagation. These eggs are scattered with the manure in the pastures, in the fodder, and in the drinking water of the animals, where they are swallowed by the cattle and develop into beef cysticerci, or *cysticercus tennicollis* in contradistinction to the *cysticercus cellulosae* of the *tænia solium.* Like the pork cysticercus, the beef cysticercus is found dispersed through all parts of the body, but observation has shown that it is found most frequently in the muscles and in the heart, rarely in the liver, kidneys, lungs, etc.

Having given this general description of *tænia medio-canellata*, I will relate the clinical history of a patient affected with this worm, under my care last year in the Medical Clinic of Florence, on whom Dr. Vanni, assistant at the clinic, desired to try *thymic acid* as a remedy.

The medicines which have been used as anthelmintics are almost without number, many of them being warranted as infallible cures. The most ancient is the *rhizome* of the *filix mas*, of which Pliny, Galen and Theophrastus have spoken in their books, and which is generally looked upon as the chief of anthelmintics although it often excites vomiting and other disturbances. It generally succeeds in expelling the *bothriocephalus latus* and *tænia solium*, but often fails with the *tænia medio-canellata*.

I do not propose to discuss the properties, advantages and defects of each remedy in detail, as that would carry me too far from my subject. Moreover, I do not consider it necessary in demonstrating the value of a new remedy to point out the real or supposed defects of the old, thus performing a thankless work of destruction. Such a course often carries us far away from the truth. Among the anthelmintics employed with more or less success we have besides *filix mas*, the *bark of the pomegranate*, the *flowers of koosso*, *kamala*, introduced into Italy in 1864 by Cantani, *turpentine*, *pumpkin seeds*, *sulphuric ether*, *ocymum basilicum*, *chloride of sodium*, and many others, without mentioning some which have at present fallen into disuse, such as the *powdered tin* and *tincture of nux vomica*, without mentioning the special remedies in vogue wherever tape-worm is found, and without including the various empirical remedies often containing the most dangerous substances such as arsenic and the other mineral poisons which are liable to cause death. Finally we will call attention to Constatt's classification of anthelmintics into mechanical and dynamic remedies. This division is not accurate, as some of the remedies act in both manners. Constatt favors us with the description of no less than fifty anthelmintics. But, fortunately, science and practice have in their glorious work of elimination, taught us to discard the dangerous remedies and to use with distrust many others.

With regard to *thymic acid* or *thymol*, we know that it is derived from one of the *labiatae*, thyme (*thymus serpillus*, *vulgaris* and *piperella*), which is common throughout France and Southern Italy. The plant contains a volatile substance, a liquid essence, called *thymine*, which is isomeric with turpentine also a tæniafuge. It is doubtless to this volatile substance that *thymol* owes its anthelmintic properties, although the tannin and a bitter principle are of importance. Thymic acid or thymol, when perfectly pure, resemble camphor, but is much more transparent and has a glassy appearance.

It is very slightly soluble in water but can be readily dissolved in alcohol, in ether, and in alkaline solutions. It has an acrid, warm, somewhat persistent bitter taste and the odor of thyme.

We know but little of its biological effects. Lewin, a few years ago, claimed that it had valuable germicidal and antiseptic properties, and was four times more powerful for those purposes, than carbolic acid. Bucholtz has recently made a comparative study of its germicidal properties and finds that a solution of 1 part to 2,000 will prevent the growth of bacteria, being surpassed only by bichloride of mercury, while benzoin, carbolic, salicylic and boric acids, creosote, the sulphates of quinine, copper and zinc and alcohol are all inferior in their antiseptic powers. The action of thymol, moreover, is not limited to micro-organisms, but affects the higher animals and man. Lewin discovered that frogs placed in weak solutions of thymol became insensible and lost the power of reflex action from mechanical and chemical excitation, while the electrical irritability of the muscles and nerves remained intact. Leucocytes are rapidly attacked by thymol and lose their motility. From the description of the properties of thymol it is not difficult to conceive that it may be usefully employed in the treatment of tape-worm, especially since the experiments of Federici have shown that it is very effective for the destruction of the *anchylostoma duoaenalis*, the parasite which produces the terrible infirmity called *Gottardo's disease*, or *miners' anæmia*. But in order to succeed with a remedy, it is not enough to know its physiological action, we must also know how to administer it posology is undoubtedly the most important and the most delicate part of the practice of medicine. This simple truth, so generally recognized by medical writers, must be particularly regarded in the use of anthelmintics, the effectiveness of which depends so largely on the mode of administration.

Dr. Vanni, in the first case of tænia treated by thymol gave three grammes (gr. xlv.) in divided doses through the day, and twenty grammes (ʒ v.) of castor oil at night, but succeeded in obtaining only five or six segments of the worm. On the following day, however, he administered six grammes, (ʒ iss.) of thymol, divided into twelve doses to be taken every fifteen minutes, and at the end of the third hour the entire worm including the head, was expelled.

I adopted the following method with my first case: In the evening I ordered twenty grammes (ʒ v.) of castor oil to be taken fasting; in the morning I prescribed eight grammes, (ʒ ii.) of thymol divided into twelve doses, one to be taken every quarter of an hour; twenty minutes after the last dose had been swallowed, another twenty grammes (ʒ v.) of castor oil were taken. A few minutes after, a tænia medio-canellata three and a half meters in length was evacuated, the head being dead. To be sure, I had no Shultze's table with me to prove that there was no movement, but I touched the worm repeatedly with a hot coal, and observed no sign of life.

It is very important during the administration of the thymic acid, to give the patient some cordial and stimulant, cognac, rum and alkermes will be found suitable. Thymol is very depressing according to the experiments of Husemann, and this will explain its action on the nervous system. Even with small doses the pulse becomes small and frequent, the respiratory movements slow, and the temperature is lowered. These effects, however, are easily and promptly counteracted by the use of stimulants.

I must call attention to another fact. Husemann and Lewin say that thymol should be administered in small doses. On account of its caustic action, they claim that there is danger of producing digestive disturbances and gastro-enteritis. They recommended two or three teaspoonfuls of a one-half per cent. solution during the day. While it is true that we can never be too prudent in the practice of medicine, these doses are too fearfully homœopathic, when we consider that eight grammes of thymic acid may be given in two hours and a half with out producing the least gastro-intestinal disturbance.

I may be criticised for offering this new anthelmintic to the profession on the ground that there is no occasion for new remedies of this class. In reply, I would state that no specific tæniafuge has yet been discovered. The number of remedies recommended prove that none are reliable. The tæniafuges have left the physician and gone to the druggist who sells his infallible vermifuges as he does his hair restorers. We have only to ask any old practitioner about the use of anthelmintics to learn that this class of remedies is exceedingly unreliable. Most of these remedies, moreover, produce grave gastro-intestinal disturbances and we are not surprised that Bamberger questioned which had done mankind most harm, the tape-worms or the tæniafuges. But the time spent in clinical researches is not wasted even if we arrive at negative results, and although my observations are as yet limited, I would respectfully recommend to the profession the use of thymic acid as a tæniafuge :

1. Because this remedy, with the exception of a depressing effect, easily counteracted, produces no disturbance of the stomach or intestines.

2. On account of the rapidity and simplicity of its action compared with other remedies, which require *courses of treatment*, divided into three periods, the preparatory, the expulsive, and the consecutive.

3. On account of the advantages it offers of being both a tæniacide, and a tæniafuge.

4. Because in case of an error of diagnosis the remedy would produce an efficient purgation and disinfection of the alimentary canal.

5. Because it is reasonable to suppose that since thymic acid succeeds in expelling the tænia medio-canellata, the worm which above all others resists the action of anthelmintics the same remedy will be of service in expelling all other varieties of flat worms.

6. Because we have, as yet, always been successful with the use of thymic acid and have probably found a real specific, which up to the present did not exist.

